# 4-Chloro-*N*-methyl­benzamide

**DOI:** 10.1107/S1600536812008641

**Published:** 2012-03-03

**Authors:** Juan Yuan, Yan-Ju Liu

**Affiliations:** a Pharmacy College, Henan University of Traditional Chinese Medicine, Zhengzhou 450008, People’s Republic of China

## Abstract

There are two mol­ecules in the asymmetric unit of the title compound, C_8_H_8_ClNO, which are linked in the crystal structure *via* N—H⋯O hydrogen bonds into chains along the *b* axis. C—H⋯O contacts also occur. The benzene ring makes dihedral angles of 5.9 (1) and 16.7 (1)°with the attached amide group in the two independent molecules.

## Related literature

For applications of the title compound and background to the synthesis, see: Lee *et al.* (2009 ▶). For bond-length data, see: Allen *et al.* (1987 ▶).
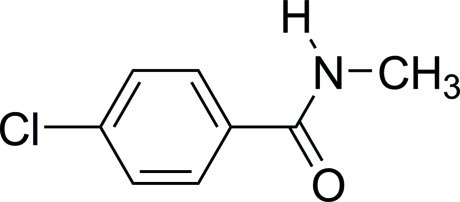


## Experimental

### 

#### Crystal data

C_8_H_8_ClNO
*M* *_r_* = 169.61
Triclinic, 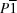

*a* = 3.9420 (8) Å
*b* = 9.2250 (18) Å
*c* = 21.864 (4) Å
α = 96.46 (3)°
β = 90.34 (3)°
γ = 90.99 (3)°
*V* = 789.9 (3) Å^3^
*Z* = 4
Mo *K*α radiation
μ = 0.42 mm^−1^
*T* = 296 K
0.20 × 0.10 × 0.10 mm

#### Data collection

Enraf–Nonius CAD-4 diffractometer
Absorption correction: ψ scan (North *et al.*, 1968 ▶) *T*_min_ = 0.921, *T*_max_ = 0.959
3079 measured reflections
2887 independent reflections
1633 reflections with *I* > 2σ(*I*)
*R*_int_ = 0.047
3 standard reflections every 200 reflections intensity decay: 1%

#### Refinement

*R*[*F* ^2^ > 2σ(*F* ^2^)] = 0.068
*wR*(*F* ^2^) = 0.188
*S* = 1.00
2887 reflections
199 parameters
2 restraints
H-atom parameters constrained
Δρ_max_ = 0.29 e Å^−3^
Δρ_min_ = −0.25 e Å^−3^

### 

Data collection: *CAD-4 Software* (Enraf–Nonius, 1985 ▶); cell refinement: *CAD-4 Software*; data reduction: *XCAD4* (Harms & Wocadlo, 1995 ▶); program(s) used to solve structure: *SHELXS97* (Sheldrick, 2008 ▶); program(s) used to refine structure: *SHELXL97* (Sheldrick, 2008 ▶); molecular graphics: *SHELXTL* (Sheldrick, 2008 ▶); software used to prepare material for publication: *SHELXTL*.

## Supplementary Material

Crystal structure: contains datablock(s) I, global. DOI: 10.1107/S1600536812008641/bq2339sup1.cif

Structure factors: contains datablock(s) I. DOI: 10.1107/S1600536812008641/bq2339Isup2.hkl

Supplementary material file. DOI: 10.1107/S1600536812008641/bq2339Isup3.cml

Additional supplementary materials:  crystallographic information; 3D view; checkCIF report

## Figures and Tables

**Table 1:** Hydrogen-bond geometry (Å, °)

*D*—H⋯*A*	*D*—H	H⋯*A*	*D*⋯*A*	*D*—H⋯*A*
N1—H1*A*⋯O2^i^	0.86	2.07	2.876 (4)	157
N2—H2*B*⋯O1^ii^	0.86	2.06	2.887 (4)	160
C5—H5*A*⋯O2^i^	0.93	2.53	3.417 (5)	159
C9—H9*A*⋯O1^ii^	0.93	2.60	3.379 (5)	142

Symmetry codes: (i) 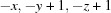; (ii) 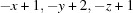.

**Fig. 1.**
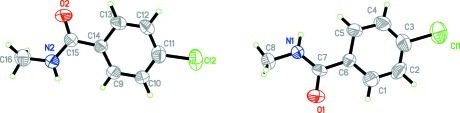
The molecular structure of (I), with the atom-numbering scheme. Displacement ellipsoids are drawn at the 50% probability level.

**Fig. 2.**
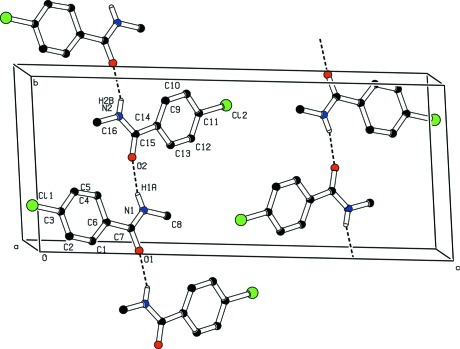
A packing diagram of (I) showing N-H..O hydrogen-bonded chains along b axis. H atoms not involved in bonding are omitted for clarity.

## supplementary crystallographic information

### Comment

Benzamide derivatives exhibit interesting biological activities such as antibacterial and antifungal effects (Lee *et al.*, 2009). We report here the crystal structure of the title compound 4-chloro-*N*-methylbenzamide, (I).

The molecular structure of (I) is shown in Fig. 1. The title compound was connected together *via* N—H···O intermolecular hydrogen bonds (Table 1), supported by a C—H···O contact, forming chains along *b* axis direction (Figure 2.).

The asymmetric unit contains two title molecules of 4-chloro-*N*-methylbenzamide. The rings of these molecules are planar with r.m.s. deviation of 0.0048 Å and 0.0034 Å. The dihedral angles of the planes A(C1—C6), B(C7/O1/N1/H1A), C(C9—C14), D(C15/O2/N2/H2B) are: A/B = 5.9 (1)°, C/D = 16.7 (1)° and A/C = 16.77 (16) °.

### Experimental

The title compound, (I) was prepared by a method reported in literature (Lee *et al.*, 2009). The crystals were obtained by dissolving (I) (0.1 g) in methanol (30 ml) and evaporating the solvent slowly at room temperature for about 8 d.

### Refinement

All H atoms were positioned geometrically and constrained to ride on their parent atoms, with C—H = 0.93 Å for aromatic H, 0.96 Å for methyl H and 0.86 Å for N—H, respectively. The *U*_iso_(H) = *xU*_eq_(C), where *x* = 1.2 for aromatic H and N—H, and *x* = 1.5 for methyl H.

### Crystal data

**Table:**

C_8_H_8_ClNO	*Z* = 4
*M**_r_* = 169.61	*F*(000) = 352
Triclinic, *P*1	*D*_x_ = 1.426 Mg m^−^^3^
Hall symbol: -P 1	Mo *K*α radiation, λ = 0.71073 Å
*a* = 3.9420 (8) Å	Cell parameters from 25 reflections
*b* = 9.2250 (18) Å	θ = 9–12°
*c* = 21.864 (4) Å	µ = 0.42 mm^−^^1^
α = 96.46 (3)°	*T* = 296 K
β = 90.34 (3)°	Block, colourless
γ = 90.99 (3)°	0.20 × 0.10 × 0.10 mm
*V* = 789.9 (3) Å^3^	

### Data collection

**Table:**

Enraf–Nonius CAD-4 diffractometer	1633 reflections with *I* > 2σ(*I*)
Radiation source: fine-focus sealed tube	*R*_int_ = 0.047
Graphite monochromator	θ_max_ = 25.4°, θ_min_ = 1.9°
ω/2θ scans	*h* = −4→4
Absorption correction: ψ scan (North *et al.*, 1968)	*k* = −11→0
*T*_min_ = 0.921, *T*_max_ = 0.959	*l* = −26→26
3079 measured reflections	3 standard reflections every 200 reflections
2887 independent reflections	intensity decay: 1%

### Refinement

**Table:**

Refinement on *F*^2^	Primary atom site location: structure-invariant direct methods
Least-squares matrix: full	Secondary atom site location: difference Fourier map
*R*[*F*^2^ > 2σ(*F*^2^)] = 0.068	Hydrogen site location: inferred from neighbouring sites
*wR*(*F*^2^) = 0.188	H-atom parameters constrained
*S* = 1.00	*w* = 1/[σ^2^(*F*_o_^2^) + (0.094*P*)^2^] where *P* = (*F*_o_^2^ + 2*F*_c_^2^)/3
2887 reflections	(Δ/σ)_max_ = 0.001
199 parameters	Δρ_max_ = 0.29 e Å^−^^3^
2 restraints	Δρ_min_ = −0.25 e Å^−^^3^

### Special details

**Table:**

Geometry. All e.s.d.'s (except the e.s.d. in the dihedral angle between two l.s. planes) are estimated using the full covariance matrix. The cell e.s.d.'s are taken into account individually in the estimation of e.s.d.'s in distances, angles and torsion angles; correlations between e.s.d.'s in cell parameters are only used when they are defined by crystal symmetry. An approximate (isotropic) treatment of cell e.s.d.'s is used for estimating e.s.d.'s involving l.s. planes.
Refinement. Refinement of *F*^2^ against ALL reflections. The weighted *R*-factor *wR* and goodness of fit *S* are based on *F*^2^, conventional *R*-factors *R* are based on *F*, with *F* set to zero for negative *F*^2^. The threshold expression of *F*^2^ > σ(*F*^2^) is used only for calculating *R*-factors(gt) *etc*. and is not relevant to the choice of reflections for refinement. *R*-factors based on *F*^2^ are statistically about twice as large as those based on *F*, and *R*- factors based on ALL data will be even larger.

### Fractional atomic coordinates and isotropic or equivalent isotropic displacement parameters (Å^2^)

**Table:**

	*x*	*y*	*z*	*U*_iso_*/*U*_eq_	
Cl1	1.2503 (4)	0.71959 (16)	1.02286 (5)	0.0971 (5)	
N1	0.7112 (8)	0.7694 (3)	0.73236 (14)	0.0536 (8)	
H1A	0.6619	0.6842	0.7424	0.064*	
O1	0.9592 (8)	0.9833 (3)	0.75794 (13)	0.0748 (9)	
C1	1.1009 (11)	0.9251 (5)	0.87664 (19)	0.0723 (13)	
H1B	1.1381	1.0195	0.8667	0.087*	
C2	1.1874 (12)	0.8941 (5)	0.9344 (2)	0.0751 (13)	
H2A	1.2858	0.9658	0.9626	0.090*	
C3	1.1290 (11)	0.7593 (5)	0.94995 (19)	0.0642 (11)	
C4	0.9934 (12)	0.6541 (5)	0.9090 (2)	0.0732 (13)	
H4A	0.9623	0.5610	0.9207	0.088*	
C5	0.8993 (12)	0.6791 (4)	0.85058 (19)	0.0659 (12)	
H5A	0.7988	0.6064	0.8231	0.079*	
C6	0.9649 (9)	0.8253 (3)	0.83385 (16)	0.0478 (9)	
C7	0.8729 (9)	0.8632 (4)	0.77223 (17)	0.0470 (9)	
C8	0.6136 (11)	0.8043 (4)	0.67242 (18)	0.0625 (11)	
H8A	0.4946	0.7224	0.6507	0.094*	
H8B	0.8125	0.8266	0.6499	0.094*	
H8C	0.4683	0.8872	0.6766	0.094*	
Cl2	0.3429 (3)	0.77031 (14)	0.52328 (5)	0.0843 (5)	
O2	−0.3948 (7)	0.5109 (3)	0.25934 (13)	0.0690 (8)	
N2	−0.3020 (8)	0.7363 (3)	0.23575 (14)	0.0575 (9)	
H2B	−0.2129	0.8209	0.2470	0.069*	
C9	−0.0638 (11)	0.8149 (4)	0.36052 (18)	0.0615 (11)	
H9A	−0.1235	0.8915	0.3386	0.074*	
C10	0.0805 (11)	0.8473 (4)	0.41801 (18)	0.0626 (11)	
H10A	0.1145	0.9435	0.4350	0.075*	
C11	0.1731 (10)	0.7327 (4)	0.44960 (18)	0.0592 (10)	
C12	0.1202 (10)	0.5922 (4)	0.42494 (18)	0.0608 (11)	
H12A	0.1841	0.5163	0.4470	0.073*	
C13	−0.0237 (10)	0.5619 (4)	0.36903 (18)	0.0593 (11)	
H13A	−0.0589	0.4652	0.3528	0.071*	
C14	−0.1232 (9)	0.6758 (3)	0.33430 (16)	0.0444 (8)	
C15	−0.2870 (10)	0.6352 (4)	0.27428 (17)	0.0504 (9)	
C16	−0.4602 (11)	0.7105 (4)	0.17627 (19)	0.0694 (12)	
H16A	−0.4403	0.7968	0.1557	0.104*	
H16B	−0.6957	0.6861	0.1809	0.104*	
H16C	−0.3507	0.6312	0.1524	0.104*	

### Atomic displacement parameters (Å^2^)

**Table:**

	*U*^11^	*U*^22^	*U*^33^	*U*^12^	*U*^13^	*U*^23^
Cl1	0.1301 (12)	0.1145 (11)	0.0491 (7)	−0.0039 (8)	−0.0234 (7)	0.0221 (7)
N1	0.076 (2)	0.0421 (17)	0.0432 (18)	−0.0096 (15)	−0.0155 (15)	0.0103 (14)
O1	0.113 (2)	0.0438 (16)	0.069 (2)	−0.0172 (15)	−0.0122 (17)	0.0171 (14)
C1	0.102 (3)	0.062 (3)	0.053 (3)	−0.020 (2)	−0.011 (2)	0.012 (2)
C2	0.099 (3)	0.072 (3)	0.052 (3)	−0.032 (2)	−0.022 (2)	0.001 (2)
C3	0.072 (3)	0.072 (3)	0.051 (3)	0.015 (2)	−0.002 (2)	0.013 (2)
C4	0.116 (4)	0.052 (2)	0.053 (3)	−0.005 (2)	−0.010 (2)	0.015 (2)
C5	0.104 (3)	0.041 (2)	0.054 (3)	−0.004 (2)	−0.013 (2)	0.0096 (17)
C6	0.061 (2)	0.0419 (19)	0.039 (2)	−0.0028 (16)	−0.0064 (17)	0.0023 (15)
C7	0.058 (2)	0.0308 (18)	0.054 (2)	0.0032 (16)	−0.0016 (17)	0.0098 (15)
C8	0.080 (3)	0.061 (2)	0.048 (2)	0.005 (2)	−0.012 (2)	0.0118 (18)
Cl2	0.1028 (9)	0.1021 (10)	0.0483 (7)	0.0030 (7)	−0.0151 (6)	0.0110 (6)
O2	0.099 (2)	0.0387 (15)	0.068 (2)	−0.0151 (14)	−0.0110 (16)	0.0061 (13)
N2	0.085 (2)	0.0421 (17)	0.046 (2)	−0.0030 (15)	−0.0068 (16)	0.0078 (14)
C9	0.093 (3)	0.041 (2)	0.052 (3)	0.002 (2)	−0.015 (2)	0.0115 (17)
C10	0.102 (3)	0.037 (2)	0.049 (2)	0.001 (2)	−0.003 (2)	0.0020 (16)
C11	0.064 (3)	0.067 (3)	0.047 (2)	0.015 (2)	0.0010 (19)	0.0052 (19)
C12	0.079 (3)	0.053 (2)	0.054 (3)	0.011 (2)	−0.008 (2)	0.0194 (19)
C13	0.080 (3)	0.042 (2)	0.057 (3)	0.0145 (19)	0.003 (2)	0.0108 (18)
C14	0.052 (2)	0.0384 (19)	0.043 (2)	0.0040 (15)	0.0077 (16)	0.0034 (15)
C15	0.064 (2)	0.044 (2)	0.044 (2)	0.0031 (17)	0.0059 (18)	0.0069 (17)
C16	0.086 (3)	0.066 (3)	0.057 (3)	−0.007 (2)	−0.022 (2)	0.013 (2)

### Geometric parameters (Å, º)

**Table:**

Cl1—C3	1.741 (4)	Cl2—C11	1.737 (4)
N1—C7	1.311 (4)	O2—C15	1.224 (4)
N1—C8	1.436 (4)	N2—C15	1.327 (4)
N1—H1A	0.8600	N2—C16	1.433 (5)
O1—C7	1.227 (4)	N2—H2B	0.8600
C1—C6	1.339 (5)	C9—C14	1.360 (5)
C1—C2	1.368 (6)	C9—C10	1.376 (5)
C1—H1B	0.9300	C9—H9A	0.9300
C2—C3	1.342 (6)	C10—C11	1.379 (5)
C2—H2A	0.9300	C10—H10A	0.9300
C3—C4	1.345 (6)	C11—C12	1.359 (5)
C4—C5	1.373 (5)	C12—C13	1.343 (5)
C4—H4A	0.9300	C12—H12A	0.9300
C5—C6	1.456 (4)	C13—C14	1.423 (5)
C5—H5A	0.9300	C13—H13A	0.9300
C6—C7	1.474 (5)	C14—C15	1.467 (5)
C8—H8A	0.9600	C16—H16A	0.9600
C8—H8B	0.9600	C16—H16B	0.9600
C8—H8C	0.9600	C16—H16C	0.9600
			
C7—N1—C8	122.2 (3)	C15—N2—C16	122.8 (3)
C7—N1—H1A	118.9	C15—N2—H2B	118.6
C8—N1—H1A	118.9	C16—N2—H2B	118.6
C6—C1—C2	122.7 (4)	C14—C9—C10	123.0 (3)
C6—C1—H1B	118.6	C14—C9—H9A	118.5
C2—C1—H1B	118.6	C10—C9—H9A	118.5
C3—C2—C1	119.4 (4)	C9—C10—C11	117.9 (4)
C3—C2—H2A	120.3	C9—C10—H10A	121.0
C1—C2—H2A	120.3	C11—C10—H10A	121.0
C2—C3—C4	120.7 (4)	C12—C11—C10	121.0 (4)
C2—C3—Cl1	119.0 (3)	C12—C11—Cl2	120.0 (3)
C4—C3—Cl1	120.2 (3)	C10—C11—Cl2	118.9 (3)
C3—C4—C5	122.5 (4)	C13—C12—C11	120.5 (4)
C3—C4—H4A	118.8	C13—C12—H12A	119.7
C5—C4—H4A	118.8	C11—C12—H12A	119.7
C4—C5—C6	116.6 (4)	C12—C13—C14	120.9 (4)
C4—C5—H5A	121.7	C12—C13—H13A	119.6
C6—C5—H5A	121.7	C14—C13—H13A	119.6
C1—C6—C5	118.1 (4)	C9—C14—C13	116.7 (4)
C1—C6—C7	121.1 (3)	C9—C14—C15	125.2 (3)
C5—C6—C7	120.8 (3)	C13—C14—C15	118.1 (3)
O1—C7—N1	120.1 (3)	O2—C15—N2	121.1 (4)
O1—C7—C6	118.8 (3)	O2—C15—C14	121.2 (3)
N1—C7—C6	121.1 (3)	N2—C15—C14	117.6 (3)
N1—C8—H8A	109.5	N2—C16—H16A	109.5
N1—C8—H8B	109.5	N2—C16—H16B	109.5
H8A—C8—H8B	109.5	H16A—C16—H16B	109.5
N1—C8—H8C	109.5	N2—C16—H16C	109.5
H8A—C8—H8C	109.5	H16A—C16—H16C	109.5
H8B—C8—H8C	109.5	H16B—C16—H16C	109.5
			
C6—C1—C2—C3	−1.4 (7)	C14—C9—C10—C11	1.1 (6)
C1—C2—C3—C4	1.4 (7)	C9—C10—C11—C12	−0.7 (6)
C1—C2—C3—Cl1	178.3 (4)	C9—C10—C11—Cl2	−177.9 (3)
C2—C3—C4—C5	−1.9 (7)	C10—C11—C12—C13	0.1 (6)
Cl1—C3—C4—C5	−178.8 (4)	Cl2—C11—C12—C13	177.3 (3)
C3—C4—C5—C6	2.2 (7)	C11—C12—C13—C14	0.2 (6)
C2—C1—C6—C5	1.7 (7)	C10—C9—C14—C13	−0.9 (6)
C2—C1—C6—C7	179.6 (4)	C10—C9—C14—C15	177.7 (4)
C4—C5—C6—C1	−2.0 (6)	C12—C13—C14—C9	0.2 (6)
C4—C5—C6—C7	−179.9 (4)	C12—C13—C14—C15	−178.4 (4)
C8—N1—C7—O1	−3.2 (6)	C16—N2—C15—O2	3.7 (6)
C8—N1—C7—C6	179.0 (3)	C16—N2—C15—C14	−179.0 (3)
C1—C6—C7—O1	8.5 (6)	C9—C14—C15—O2	−164.7 (4)
C5—C6—C7—O1	−173.6 (4)	C13—C14—C15—O2	13.9 (5)
C1—C6—C7—N1	−173.6 (4)	C9—C14—C15—N2	18.0 (6)
C5—C6—C7—N1	4.3 (5)	C13—C14—C15—N2	−163.5 (3)

### Hydrogen-bond geometry (Å, º)

**Table:**

*D*—H···*A*	*D*—H	H···*A*	*D*···*A*	*D*—H···*A*
N1—H1*A*···O2^i^	0.86	2.07	2.876 (4)	157
N2—H2*B*···O1^ii^	0.86	2.06	2.887 (4)	160
C5—H5*A*···O2^i^	0.93	2.53	3.417 (5)	159
C9—H9*A*···O1^ii^	0.93	2.60	3.379 (5)	142

Symmetry codes: (i) −*x*, −*y*+1, −*z*+1; (ii) −*x*+1, −*y*+2, −*z*+1.
